# Effects of a Home-Based Physical Activity Programme on Blood Biomarkers and Health-Related Quality of Life Indices in Saudi Arabian Type-2 Diabetes Mellitus Patients: Protocol for a Randomised Controlled Trial

**DOI:** 10.3390/ijerph19084468

**Published:** 2022-04-07

**Authors:** Jonathan Sinclair, Hussein Ageely, Mohamed Salih Mahfouz, Abdulrahman Ahmed Hummadi, Hussain Darraj, Yahia Solan, Robert Allan, Lindsay Bottoms

**Affiliations:** 1 Research Centre for Applied Sport, Physical Activity and Performance, Faculty of Allied Health and Wellbeing, School of Sport & Health Sciences, University of Central Lancashire, Preston PR1 2HE, Lancashire, UK; rallan1@uclan.ac.uk; 2 Department of Family and Community Medicine, Faculty of Medicine, Jazan University, Jazan 82911, Saudi Arabia; hageely@gmail.com (H.A.); mm.mahfouz@gmail.com (M.S.M.); 3 Jazan Diabetes and Endocrinology Center, Jazan 82723, Saudi Arabia; drhummadi@hotmail.com (A.A.H.); dr.hadarraj@gmail.com (H.D.); dr.solan@gmail.com (Y.S.); 4 Centre for Research in Psychology and Sport Sciences, School of Life and Medical Sciences, University of Hertfordshire, Hatfield AL10 9AB, Hertfordshire, UK; l.bottoms@herts.ac.uk

**Keywords:** physical activity, diabetes mellitus, Saudi Arabia, HbA1c, exercise

## Abstract

The Kingdom of Saudi Arabia is renowned for its high incidence of type-2 diabetes mellitus, with a prevalence rate of around 33%, which is expected to increase to 45.8% by 2030. Engagement in regular physical activity has been shown to significantly attenuate non-communicable diseases including type-2 diabetes. However, the overall rate of physical inactivity among Saudi Arabian adults is currently 80.5%, owing to time pressures, high-density traffic, poor air quality, lack of suitable exercise places/sports facilities, lack of social/friends support, gender, cultural barriers, low self-confidence, lack of time and environmental factors. Previous analyses have shown that home-based activity interventions can be effective. Therefore, given the aforementioned barriers to physical activity in Saudi Arabia; a home-based physical activity may be an ideal solution in type-2 diabetic patients. This manuscript describes the study protocol for a randomized control trial, examining the effects of a home-based physical activity intervention in Saudi Arabian adults with type-2 diabetes. The study will recruit 62 individuals with type-2 diabetes from the Jazan region of the Kingdom of Saudi Arabia, who will be individually randomized to either a physical activity or control group. This 24-week investigation will involve 12-weeks of physical activity in the physical activity group and feature three examination points i.e., baseline, 12-weeks and 24-weeks (follow-up). The primary study outcome is the between-group difference in blood HbA1c levels relative to controls. Secondary outcomes measures will be between-group differences in anthropometric, blood lipid, physical fitness, and patient-reported quality of life outcomes pertinent to type-2 diabetes. Statistical analysis will be conducted on an intention-to-treat basis. The trial has been granted ethical approval by Jazan University, Health Research Ethics Committee (REF: 2177) and formally registered as a trial (NCT04937296). We expect dissemination of the study findings from this investigation to be through publication in a leading peer-reviewed journal.

## 1. Introduction

Globally, type-2 diabetes represents a highly prevalent chronic disease that places a significant burden on life and healthcare costs [1]. In 2017, the International Diabetes Federation indicated that there were >450 million individuals with type-2 diabetes worldwide [2], with numbers projected to rise to 693 million by 2045. As such, type-2 diabetes has been dubbed the 21st century’s primary global healthcare challenge, by the World Health Organization (WHO) and the International Diabetes Federation [3].

Type-2 diabetes is characterized by insensitivity to insulin, declining insulin production and eventual pancreatic beta-cell failure [4], leading to reductions in glucose transport into the liver [1]. The condition is associated with a multifactorial aetiology that comprises both genetic and modifiable lifestyle factors [5]. Lifestyle measures, including physical activity, are key factors for the prevention and self-management in patients with type-2 diabetes [6]. Importantly, enhanced physical activity levels have long been considered a cornerstone for both type-2 diabetes prevention and management [7].

Owing to increasing levels of economic progression, urbanization and negative lifestyle alterations, the highest prevalence of type-2 diabetes is in the Middle East and North Africa [8]. In the UK, the reported prevalence of type-2 diabetes is reported as being 5.26%, yet in Saudi Arabia, a recent review study reported that the prevalence is much higher, at around 33%, and it is expected to increase to 45.8% by 2030 [9]. The WHO has recently ranked Saudi Arabia as having the second highest rate of diabetes in the Middle East (7th highest in the world) with an estimated population of 7 million living with diabetes and more than 3 million with pre-diabetes [10]. Concerningly, recent studies from Saudi Arabia revealed that healthcare costs associated with diabetes have risen by 500% in the last 10 years [11], and that diabetes directly costs around 13.9% of the total health expenditure [12]. Based on expected population growth and the aforementioned increase in diabetes prevalence it is expected that costs will triple by 2030 [13]. Therefore, it is essential that research designed to improve health/quality of life outcomes in type-2 diabetic patients be conducted in Saudi Arabia, to decrease the social and personal costs of this disease.

Physical activity is a renowned health-enhancing modality [14]. According to WHO, lack of physical activity is among the primary risk factors for non-communicable diseases and total mortality [15]. Whilst a lack of physical activity accounts for 9% of premature deaths [16], it has been shown to significantly attenuate non-communicable diseases including type-2 diabetes [17]. The WHO introduced a global initiative to attenuate inactivity levels [18]; however, concerningly recent analyses show that such enterprises are operational in only 56% of WHO member countries [19]. During the past several decades, Saudi Arabia has witnessed significant economic and technological growth that has mediated negative lifestyle alterations [20]. The overall rate of physical inactivity in Saudi Arabian adults was 80.5% [14,15], making a lack of physical activity a major concern.

Whilst it is abundantly clear that the Saudi Arabian population needs to become more physically active in order to control the rising incidence of non-communicable diseases, the process of enhancing engagement with physical activity remains a significant challenge. Possible barriers to physical activity in Saudi Arabia cited in the current literature are lack of time, high-density traffic, poor air quality, lack of suitable exercise places/sports facilities, lack of friends/social support, gender (i.e., being female), cultural barriers, low self-confidence, lack of time and environmental factors (i.e., high temperature outdoors) [21,22,23,24].

Previous analyses have shown that physical activity can be undertaken at home [25] and that home-based activity interventions can be effective [26]. With public modesty being extremely important in Islamic countries, allied to a lack of suitable exercise locations, traffic and poor air quality, this indicates that there are several significant barriers to physical activity engagement in Saudi Arabia. Therefore, a home-based physical activity programme with a social component individualized for males and females may present an ideal solution to increase physical activity and exercise participation in Saudi Arabia. There is, however, no available information concerning the efficacy of a home-based exercise program for type-2 diabetic patients. Accounting for the incidence of type-2 diabetes, rate of physical inactivity in Saudi Arabia and the specific barriers to physical activity in this region; a home-based approach to delivering physical activity appears to be strongly warranted.

### 1.1. Aims

The purpose of this research project is to undertake a randomized control trial, examining the effects of a 12-week home-based physical activity programme in Saudi Arabian adults with type-2 diabetes. The primary objective of this randomized trial is to examine the influence of the physical activity programme on blood HbA1c levels relative to controls. Its secondary objectives are to determine whether the intervention impacts other anthropometric, blood biomarker, physical fitness, and patient-reported quality of life outcomes pertinent to type-2 diabetes.

### 1.2. Hypotheses

In relation to the primary outcome, the physical activity intervention will mediate reductions in HbA1c levels compared to the control group. Furthermore, for the secondary outcomes body mass, body mass index, blood pressure, physical fitness will improve as a function of the physical activity intervention compared to control.

## 2. Materials and Methods

### 2.1. Study Design and Setting

This investigation is described according to the updated guidelines for reporting parallel group randomized trials [27]. The trial will take place at the Jazan Diabetes and Endocrinology Center, located in the Jazan region of southwestern Saudi Arabia. The population of Jazan is relatively homogeneous, with inhabitants habitually sharing the same language, ethnicity and religion [28].

**Figure 1:**
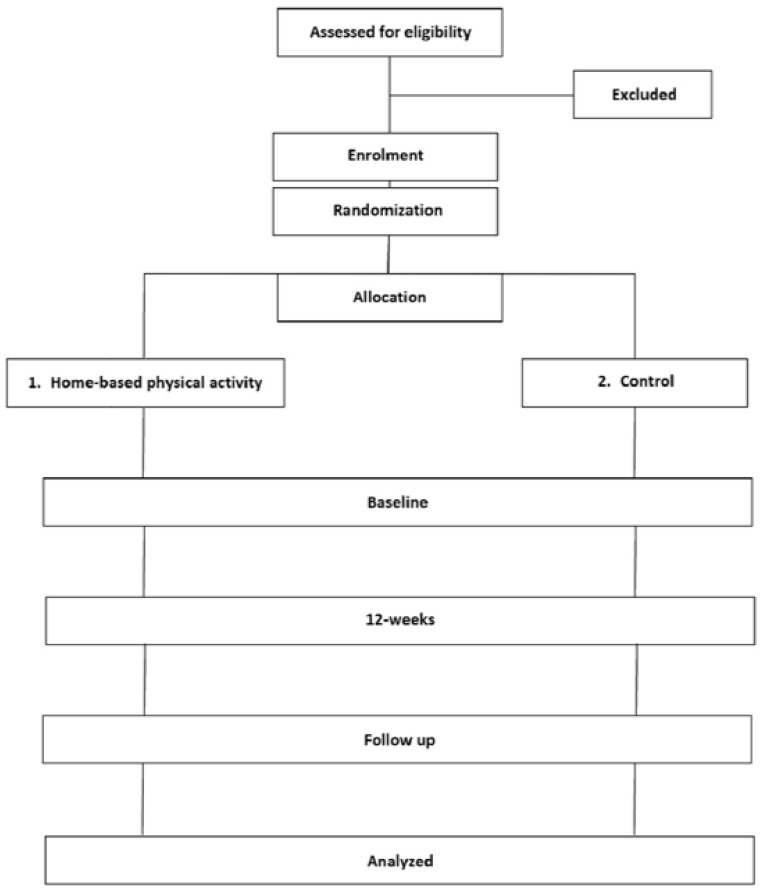
Consort diagram showing the study design.

This investigation represents a 24-week (in total) parallel randomized controlled trial (Figure 1). After screening for eligibility and enrolment, participants will then be individually randomized by a computer program (Random Allocation Software) to 12-weeks of: (1). Home-based physical activity or (2). Control group. Primary and secondary outcome variables, as described in detail below, will be assessed at baseline, 12-weeks and at follow-up (24-weeks). In agreement with previous trials of type-2 diabetes management, the primary outcome measure will be the between-group difference in HbA1c [29,30]. Secondary outcome measures will be between-group differences in anthropometric, blood pressure, resting heart rate, blood biomarkers, physical fitness, and patient-reported outcome indices.

### 2.2. Inclusion Criteria

Inclusion criteria will be a clinically established diagnosis of type-2 diabetes for at least 12 months, previously sedentary adults, aged over 18 years and capacity to provide informed consent.

### 2.3. Exclusion Criteria

Exclusion criteria will be cognitive impairment precluding consent or participation, pregnancy, additional medical conditions that prevent safe physical activity (e.g., severe arthritis or advanced heart failure) and enrolment in any other clinical trial designed to influence type-2 diabetes symptoms.

### 2.4. Sample Size

Power calculations were performed for the primary outcome variable i.e., the between groups difference in HbA1c. An a priori power analysis was undertaken with a significance level of 5% and 80% power, based on previous HbA1c values at baseline and at 12-weeks (of a longer duration trial) following a physical activity intervention in patients with type-2 diabetes [29]. Taking into account an expected loss to follow-up rate of 20%, this revealed that 31 participants would be required in each trial arm with a total N of 62.

### 2.5. Participants and Recruitment

This study will be conducted with patients attending the Jazan Diabetes and Endocrinology Center. Interested individuals will be able to contact the research team for further study information and to ask any questions associated with participation in the study. Participants will be invited to attend an eligibility, enrolment and familiarization session at the Jazan Diabetes and Endocrinology Center. Participants will be supported in their travel costs for attendance at the Diabetic Center during the course of this investigation. Written informed consent will be obtained from those willing to take part. All participants will be advised to maintain their previous routine medications and diets. Furthermore, those in the control group will be instructed to maintain their present lifestyle until the end of the project.

### 2.6. Home Based Physical Activity and Control Group Intervention

Participants assigned to the intervention group will be asked to perform resistance exercises 3-times a week on alternating days for 12-weeks. Each session will begin with a 5 min warmup and end with a 5 min cool down. Exercises will be performed with a TheraBand and will target all major muscle groups (See Table 1). The exercises will comprise squat, lunge, press-up, cross body reach, reverse fly, lateral raise, biceps curl, triceps extension, frontal raise, and bridge. During the first 4-weeks the participants will complete 2 sets (with 2 min rest between sets) of 10–12 repetitions of all exercises (resting for 30 s between each TheraBand exercise). This will progress to 15 repetitions during weeks 4–8. Finally, in weeks 9–12, they will perform 3 sets of 10–15 repetitions. A research nurse will supervise the first exercise session in week 1 to demonstrate the exercises to participants, then supervise the second exercise session of week 1 to ensure the participant is performing the exercise correctly. They will then supervise the final exercise sessions of weeks 4 and 8 to determine whether the participant can progress to the next stage of the programme. These supervised sessions will be completed either remotely via a video call or in person at the Diabetic Center. Immediately prior to and preceding each exercise session, capillary blood samples will be collected by finger-prick using a disposable lancet after cleaning with a 70% ethanol wipe. Capillary glucose levels (mmol/L) will be obtained using a handheld analyser in order to monitor blood glucose levels and prevent hypoglycaemia.

In addition to the resistance exercises, the participants will perform aerobic exercises. They will be asked to download an Arabic smartphone steps application to record the number of steps on day 1 of the intervention (assuming that on this day, they complete their normal daily activities as they would do most days of the week) and participants who do not possess the required phone technology will be provided with a pedometer. They will then be asked to add 2000 steps on to their daily steps; this amount will then become their daily step goal. Once they have reached this goal on 4 out of 5 days, they will be asked to increase their step goal by 500 (See Table 2). The participants will be assigned to a WhatsApp group (single sex) of up to nine people where they will be reminded to go out and walk where they can motivate one another. All participants in the intervention group will receive their usual care as well as the standard lifestyle education programme which is individualised to their needs offered by the Diabetic Center. The education programme consists of an appointment with a consultant who will provide information about changing their lifestyle and activity levels. Following this, the participants will attend two visits to the Medical Education and Nutrition clinics where they will be provided with information and support materials on physical activity.

### 2.7. Control Group

The control group will not receive any physical activity instructions; however, they will be randomised into groups of up to 9 people and be allocated to a WhatsApp group (to control for any social interaction). The participants will receive usual care as well as the lifestyle education programme described previously.

### 2.8. Data Collection

All measurements will be performed at the Jazan Diabetes and Endocrinology Center and will be undertaken in an identical manner on three occasions i.e., baseline, 12-weeks, and follow-up.

### 2.9. Demographic and Health Information

In accordance with the consensus statement for the investigation of type-2 diabetic patients [30], age, years since diagnosis, sex, race, smoking status, marital status, number of children, diabetes treatment, blood pressure-lowering therapy and educational level will be obtained by self-report at baseline.

### 2.10. Anthropometric Measurements

Anthropometric measures of body mass (kg) and stature (m) (without shoes) will be used to calculate body mass index (kg/m^2^). Stature will be measured using a stadiometer and mass will be measured using standard weighing scales. Finally, waist circumference will be measured at the midway point between the inferior margin of the last rib and the iliac crest and hip circumference around the pelvis at the point of maximum protrusion of the buttocks, without compressing the soft tissues [31]; allowing the waist-to-hip ratio to be quantified.

### 2.11. Blood Pressure and Resting Heart Rate

Blood pressure and resting heart rate measurements will be undertaken in an up-right seated position. Both peripheral measures of systolic and diastolic blood pressure and resting heart rate will be measured via a non-invasive, automated blood pressure monitor, adhering to the recommendations specified by the European Society of Hypertension [32]. Three readings will conducted, each separated by a period of 1-min [33], and the mean of the last two readings used for analysis.

### 2.12. Blood Biomarkers

At baseline, after the 12-week intervention period and at follow-up, the levels of HbA1c (primary outcome), fasting glucose and blood lipid profiles will be measured in accordance with the Nano et al. [30] consensus statement. Whole blood samples (12 mL) will be collected from the antecubital vein directly into blood collection tubes coated with ethylenediaminetetraacetic acid (EDTA). Immediately following collection, the tubes will then be centrifuged at 2000 rpm for 10 min at 4 °C. Cell-free plasma will then be removed and stored at −20 °C until analysis. Twenty-four-hour blood glucose monitoring will occur using the Libre system [34], during weeks 1 and 2 and weeks 11 and 12. This will provide gold-standard information regarding blood glucose control and important information about postprandial as well as post-exercise blood glucose responses [35].

### 2.13. Physical Fitness

Physical activity is the intervention modality and low cardiorespiratory fitness is significantly associated with impaired fasting glucose and an independent predictor of all-cause mortality in type-2 diabetes [36]. Therefore, to assess the physical activity intervention itself and also determine the mechanisms responsible for any alterations in either primary/secondary outcomes physical fitness (mL·kg·min^−1^) will be examined using the Chester step test at the timepoints outlined above [37].

### 2.14. Patient-Reported Outcome Measures

In further accordance with the consensus statement of Nano et al. [30], two generic and one diabetes-specific tool will be utilized at baseline, after the 12-week intervention period and at follow-up (translated in Arabic): The five-item WHO Well-Being Index (WHO-5), the Patient Health Questionnaire-9 (PHQ-9), and the Problem Areas in Diabetes (PAID) scale. In addition, as type-2 diabetes is associated with higher incidence of sleep disorders [38], sleep quality will be examined using the Pittsburgh Sleep Quality Index (PSQI) at baseline, after the 12-week intervention period and at follow-up. The WHO-5 and PHQ-9 have been shown to be valid and reliable screening tool for depression and as outcome measures in clinical trials [39,40]. Similarly, the PAID scale has been shown to be a valid, reliable, and sensitive research tool in patients with diabetes [41] and the PSQI has excellent test–retest reliability, specificity and validity in research and clinical settings [42]. Finally, changes in participants self-reported physical activity levels will be explored using the International Physical Activity Questionnaire short form (IPAQ-SF) [43].

### 2.15. Data Management

The collection and storage of data will adhere to the standard requirements of the UK Data Protection Act 2018. Data will be entered onto electronic spreadsheets, and then stored on a secure university server using Microsoft OneDrive(Microsoft, Washington, DC, USA). All data will be treated confidentially and anonymized for evaluation. Hard copies of data and documents will be kept in a locked and secure filing cabinet for the duration of the study. Following completion of the study, data will be transferred to the University of Central Lancashire Research Data Archive (CLOK), where it will be kept for 5-years. Hard copies will be disposed of confidentially and electronic data deleted after this period of time.

### 2.16. Statistical Analysis

All experimental data will be continuous and will therefore be presented as mean and 95% CIs. Statistical analysis of all baseline variables will be conducted to compare the two groups at baseline using linear mixed models, with group modelled as a fixed factor and random intercepts by participants. All analyses of the intervention-based data will be performed on an intention to treat basis and all randomized participants will be included in the final analysis as far as data collected will allow. Furthermore, in order to determine the effects of the intervention on all of the outcome measures, differences between the two groups will be examined using linear mixed models (at both 12-weeks and follow-up) with each group modelled as a fixed factor and random intercepts by participants adopted, adjusted for baseline values modelled as a continuous fixed covariate. For linear mixed models the mean difference (*b*), t-value and 95% confidence intervals of the difference will be presented. Finally, changes from baseline to follow-up in HbA1c will be used to create a binary variable i.e., improve/did not improve. Pearson chi-square tests of independence will be used to undertake bivariate cross-tabulation comparisons between the two trial groups, specifically to test differences in the number of participants who exhibited improvements in HbA1c. Chi-square probability values will be calculated using Monte Carlo simulation. All analyses will be conducted using SPSS v27 (IBM, SPSS, New York, NY, USA), and statistical significance accepted at the *p* ≤ 0.05 level.

### 2.17. Ethics and Dissemination

This study has been granted ethical approval by the University of Jazan, Health Research Ethics Committee (REF: 2177) and formally registered as a trial (NCT04937296). Any required alterations to the experimental protocol will be sent for re-review/approval by the research ethics committee and amended at the trial registry. Participants who express a desire to view a summary of the trial findings will be provided with such information when the data have been analysed. Dissemination of the study findings from this investigation will be through publication in a leading peer-reviewed journal and presentation at both national and international scientific conferences.

## 3. Conclusions

The proposed randomized controlled trial will explore the effects of a 12-week home-based physical activity programme in Saudi Arabian adults, on outcomes pertinent to the aetiology of type-2 diabetes mellitus and its comorbidities. Considering the high incidence of this condition in the Kingdom of Saudi Arabia, this trial may provide important clinical information regarding the non-pharmacological management of type-2 diabetes.

## Figures and Tables

**Table 1:** Resistance exercises.

Stage	Resistance Exercises	Duration	Equipment
1 (weeks 1–4)	Squat Lunge Press-Up Reverse Fly Lateral Raise Biceps Curl Triceps Extension Frontal Raise Bridge	Rest for 30 s between each type of TheraBand exercise. Rest for 2 min before repeating the stage again. Sets: 2, Repetitions: 10–12	Latex free resistance band
2 (weeks 5–8)		Rest for 30 s between each type of TheraBand exercise. Rest for 2 min before repeating the stage again. Sets: 2, Repetitions: 15	Latex free resistance band
3 (weeks 9–12)		Rest for 30 s between each type of TheraBand exercise. Rest for 2 min before repeating the stage again. Sets: 3, Repetitions: 10–15	Latex free resistance band

**Table 2:** Example Training Programme.

Sunday	Monday	Tuesday	Wednesday	Thursday	Friday	Saturday
Resistance Exercise		Resistance Exercise		Resistance Exercise		
Walking (+2500 over daily step count)	Walking (+2500 over daily step count)	Walking (+2500 over daily step count)	Walking (+2500 over daily step count)	Walking (+2500 over daily step count)	Walking (+2500 over daily step count)	Walking (+2500 over daily step count)

## Data Availability

Not applicable.
